# Posterior Reversible Encephalopathy Syndrome in a Postpartum Patient With Eclampsia

**DOI:** 10.7759/cureus.114755

**Published:** 2026-08-18

**Authors:** Umniyah Abu-Nayla, Noor Alabdi

**Affiliations:** 1 Obstetrics and Gynecology Residency Program, Dubai Health Authority, Dubai, ARE

**Keywords:** eclampsia, hypertensive emergency, neuroimaging, posterior reversible encephalopathy syndrome (pres), postpartum seizures, pregnancy-induced hypertension (pih), pres, vasogenic edema

## Abstract

Posterior reversible encephalopathy syndrome (PRES) is a neurological emergency commonly associated with hypertensive disorders of pregnancy, particularly preeclampsia and eclampsia. It is characterized by vasogenic edema predominantly affecting the parieto-occipital regions of the brain and may present with seizures, visual disturbances, altered mental status, and severe headache. Early recognition and prompt management are essential to prevent permanent complications. We report the case of a 30-year-old postpartum woman with pregnancy-induced hypertension complicated by eclampsia who developed severe headache, blurred vision, and recurrent generalized tonic-clonic seizures following a cesarean section delivery at 37 weeks and 5 days of gestation. Computed tomography (CT) of the brain demonstrated bilateral parieto-occipital hypoattenuation with additional involvement of the caudate nuclei and posterior external capsules. The radiological impression was that the findings were consistent with PRES. The patient was managed with antihypertensive therapy, magnesium sulfate, anticonvulsants, ventilatory support, and intensive care monitoring, resulting in gradual clinical neurological improvement. This case highlights the diagnostic challenge posed by an atypical and relatively extensive distribution of CT abnormalities in postpartum eclampsia, particularly when MRI and follow-up neuroimaging are unavailable for definitive lesion characterization and confirmation of radiological reversibility.

## Introduction

Posterior reversible encephalopathy syndrome (PRES) is a clinicoradiological condition characterized by acute neurological symptoms and vasogenic edema that predominantly affects the parieto-occipital regions of the brain [1-3]. Patients commonly present with headache, seizures, visual disturbances, and altered mental status, while focal neurological deficits may occur less frequently [1-3]. PRES has been associated with acute hypertension, renal insufficiency, autoimmune disease, immunosuppressive therapy, preeclampsia, and eclampsia [2-4].

Although its pathophysiology is not completely understood, the principal proposed mechanisms include impaired cerebral autoregulation during acute hypertension and endothelial dysfunction, resulting in disruption of the blood-brain barrier and the development of vasogenic edema [3,4]. Prompt recognition and treatment are important because PRES is not always completely reversible and may be complicated by cerebral infarction, intracranial hemorrhage, persistent neurological impairment, or death [3,4].

We report a case of PRES in a postpartum patient with pregnancy-induced hypertension complicated by eclampsia, in whom computed tomography (CT) demonstrated bilateral posterior abnormalities with additional involvement of deep cerebral structures. The educational significance of this case lies in the atypical and relatively extensive distribution of the CT abnormalities and the diagnostic challenge encountered in the absence of confirmatory MRI or follow-up neuroimaging.

## Case presentation

A 30-year-old primigravida underwent cesarean section delivery at 37 weeks and 5 days of gestation at a private hospital due to pregnancy-induced hypertension, gestational diabetes, and transverse fetal lie, as documented in her discharge summary. She was discharged on postoperative day 3 with oral antihypertensive medications; however, the exact names of the medications were unknown to her husband.

Shortly after discharge, the patient developed severe headache and blurred vision, followed by recurrent generalized tonic-clonic seizures. On presentation, her blood pressure was 163/110 mmHg, pulse rate was 110 beats/minute, and oxygen saturation was 92% on room air. Intravenous labetalol 20 mg was administered for blood pressure control. Despite receiving oxygen supplementation and a 4 g intravenous loading dose of magnesium sulfate, she continued to experience recurrent seizures. Intravenous midazolam was administered, followed by the initiation of a propofol infusion for seizure control. Due to persistent seizure activity and the need for airway protection, she was intubated and transferred to the intensive care unit, where magnesium sulfate was continued as a maintenance infusion.

Laboratory investigations are summarized in Table 1. Initial investigations demonstrated hypokalemia, metabolic acidosis, mild thrombocytosis, mildly elevated serum uric acid, albuminuria and aspartate aminotransferase, while serum creatinine was within normal limits.

**Table 1:** Laboratory investigations on presentation. ABG: arterial blood gas; AST: aspartate aminotransferase.

Laboratory test	Result	Reference range
Potassium in ABG	2.6 mmol/L	3.4–5.0 mmol/L
pH in ABG	7.19	7.35–7.45
Bicarbonate	18.9 mmol/L	20–28 mmol/L
Platelet count	447 ×10⁹/L	150–400 ×10⁹/L
AST	38 U/L	0-32 U/L
Creatinine	0.8 mg/dL	0.5–0.9 mg/dL
Uric acid	6.7 mg/dL	2.4–5.7 mg/dL
Urine microalbumin/creatinine ratio	100.5 mg/g	<20 mg/g

**Figure 1:**
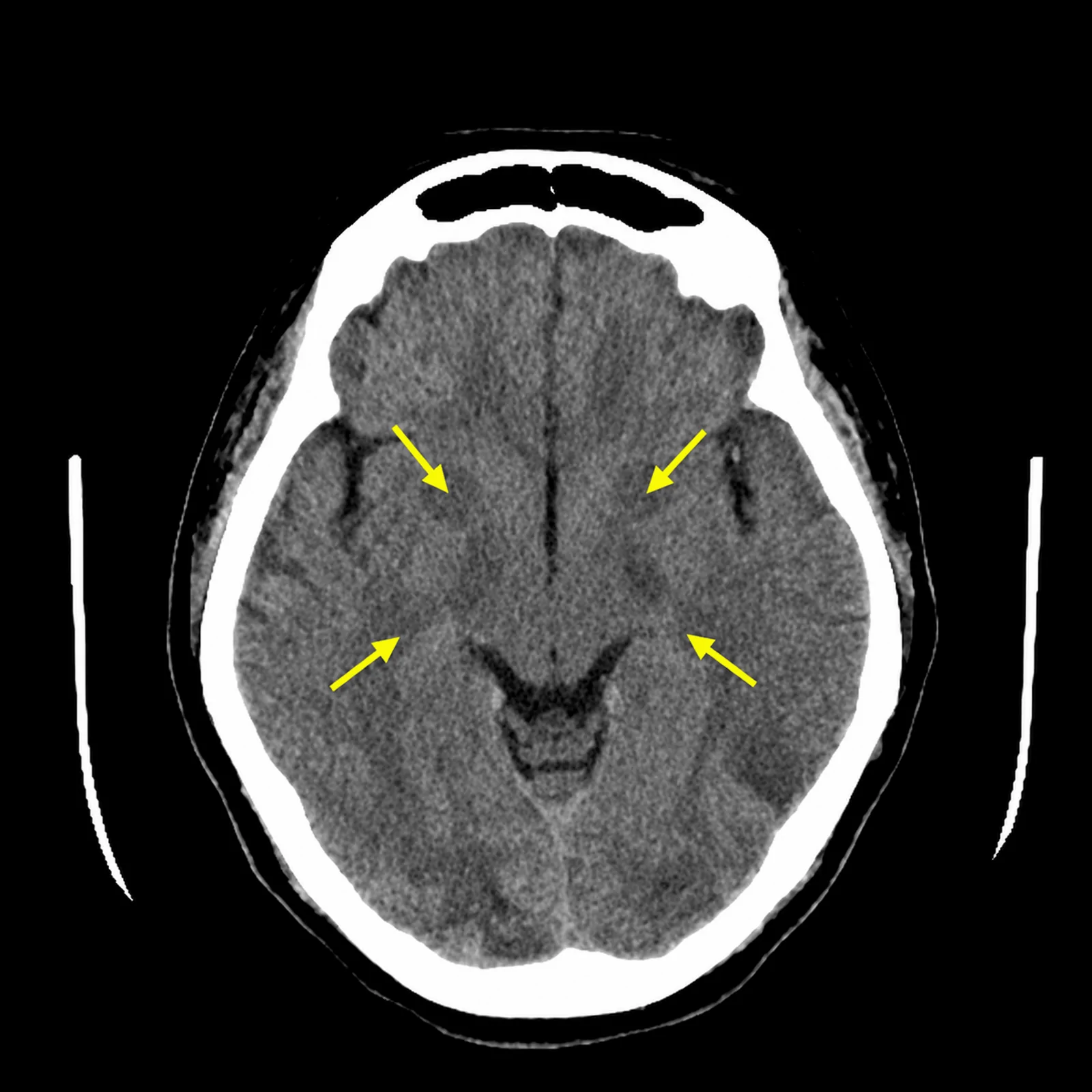
Axial non-contrast CT of the brain demonstrating hypoattenuation involving the caudate nuclei and posterior external capsules, representing the central component described in the radiology report. CT: computed tomography.

**Figure 2:**
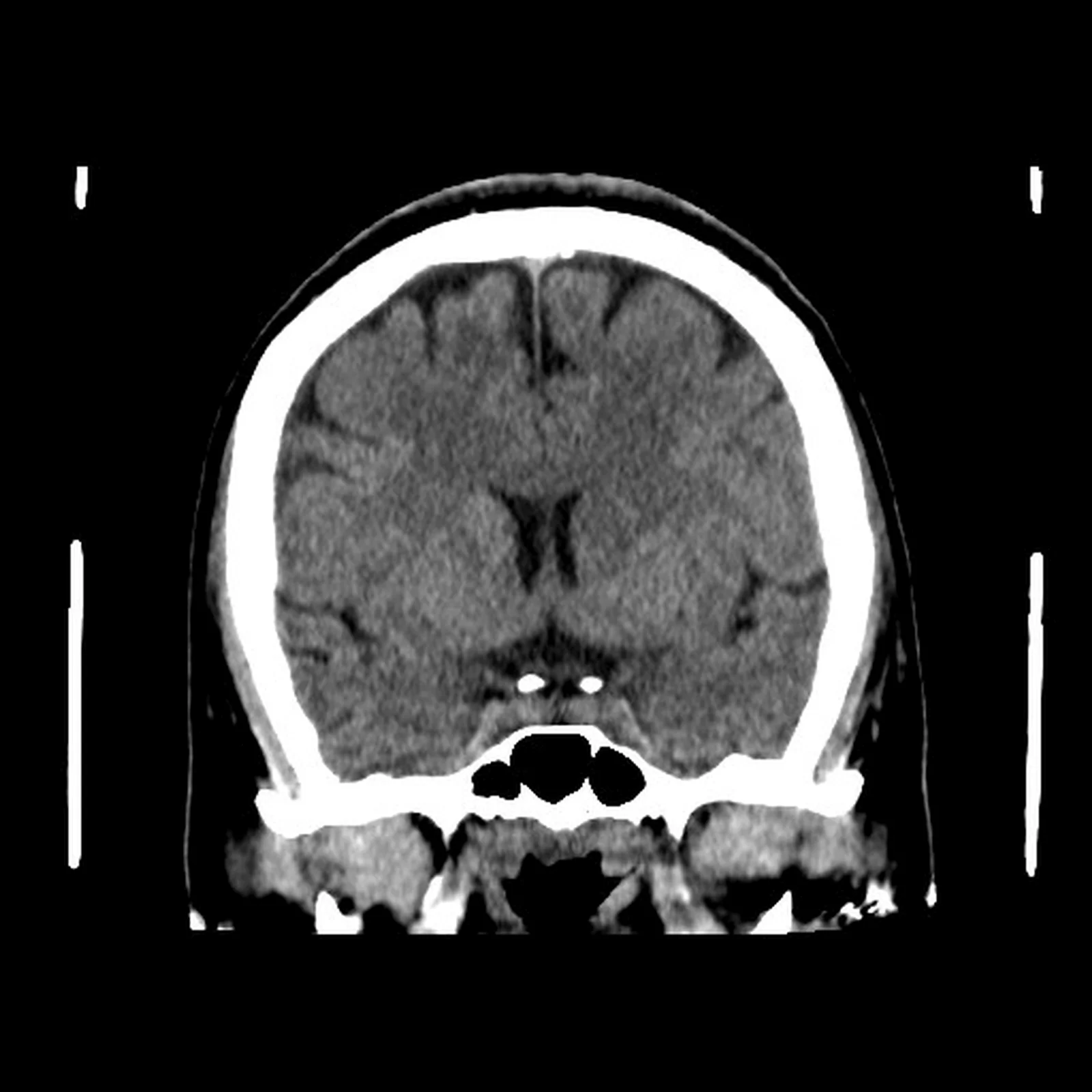
Coronal CT showing bilateral parieto-occipital hypoattenuation. CT: computed tomography.

Urgent non-contrast CT of the brain demonstrated bilateral parieto-occipital hypoattenuation with involvement of the posterior left temporal lobe. The formal radiology report also described hypoattenuation involving the left caudate nucleus, partial involvement of the posterior body of the right caudate nucleus, and minimal bilateral posterior external capsule involvement. The radiological impression was that the findings were consistent with PRES. No MRI or follow-up CT was performed; therefore, diffusion-weighted imaging (DWI)/apparent diffusion coefficient (ADC) characterization and radiological reversibility could not be assessed (Figures 1, 2).

The patient was managed with antihypertensive therapy, magnesium sulfate infusion, anticonvulsant therapy, ventilatory support, correction of electrolyte abnormalities, and close neurological monitoring in the intensive care unit. Following clinical improvement, she was extubated and transferred to the general ward after three days. She remained seizure-free, with gradual neurological recovery and stabilization of blood pressure. After a total hospital stay of 10 days, she was discharged on hydralazine 25 mg once daily, amlodipine 5 mg twice daily, and labetalol 400 mg four times daily. Outpatient follow-up was arranged; however, the patient did not attend subsequent clinic appointments.

## Discussion

PRES is a rare, potentially life-threatening clinicoradiological syndrome characterized by headache, seizures, altered mental status, and visual disturbances. Seizures, which are usually generalized tonic-clonic in nature, are often the presenting symptoms [1]. Hinchey et al. first described PRES in 1996 as a reversible syndrome caused by vasogenic edema predominantly affecting the occipitoparietal regions of the cerebral hemispheres, although atypical involvement of the brainstem, cerebellum, and other cerebral regions has also been reported [2]. Additional conditions associated with PRES include hemolytic uremic syndrome, the use of cytotoxic and immunosuppressive medications, blood transfusions, acute or chronic renal disease, and electrolyte disturbances [3]. The prevalence of PRES among patients with eclampsia has been reported to range from 51.4% to 97.9% [4]. Among patients diagnosed with preeclampsia, the reported incidence ranges between 10.5% and 19.8% [5].

Magnetic resonance imaging (MRI) remains the most sensitive imaging modality for the diagnosis of PRES because of its superior ability to detect vasogenic edema. Typical imaging findings include vasogenic edema predominantly involving the parieto-occipital regions of the brain [6]. Neuroimaging findings consistent with PRES were identified in 98% of the 47 patients with eclampsia in the study conducted by Brewer et al. [7]. Similarly, another study demonstrated MRI findings consistent with PRES in 92% of patients with eclampsia compared with only 19% of patients with preeclampsia and other neurological conditions [8].

In our patient, non-contrast CT demonstrated bilateral parietal and occipital hypoattenuation with additional involvement of the posterior left temporal lobe. The formal radiology report additionally described hypoattenuation involving the left caudate nucleus, partial involvement of the posterior body of the right caudate nucleus, and minimal bilateral posterior external capsule involvement. The radiological impression was that the findings were consistent with PRES. Although the clinical context of postpartum hypertension and eclampsia together with the radiological impression favored PRES, alternative or overlapping neurological processes could not be completely excluded in the absence of MRI with DWI and ADC assessment. The absence of MRI also limited further characterization of the CT abnormalities and the exclusion of possible cytotoxic or ischemic changes. No MRI or follow-up CT was performed; therefore, radiological reversibility could not be confirmed.

PRES generally carries a favorable prognosis, with most patients achieving complete or near-complete neurological recovery following appropriate management [9]. However, delayed diagnosis or treatment may result in permanent neurological deficits secondary to cerebral infarction, intracranial hemorrhage, or transtentorial herniation, potentially leading to death [10]. Initial management should focus on prompt blood pressure control, seizure management, maintenance of adequate oxygenation, correction of metabolic abnormalities, and supportive care [11]. Magnesium sulfate remains an important component of treatment in patients with eclampsia-associated PRES because of its anticonvulsant properties. Early administration has been associated with improved neurological recovery and may reduce the risk of persistent neurological injury [12].

## Conclusions

PRES should be considered in postpartum patients presenting with hypertension, seizures, and other acute neurological symptoms. CT may demonstrate abnormalities suggestive of PRES, including typical posterior and less common deep cerebral involvement; however, CT alone cannot definitively characterize the underlying edema or exclude alternative neurological processes. MRI with DWI and ADC assessment provides important additional characterization when available. Although our patient demonstrated clinical neurological recovery following blood pressure control, seizure management, and supportive care, radiological reversibility could not be established because neither MRI nor follow-up CT was performed.
